# Comprehensive Treatment of Severe Periodontal and Periimplant Bone Destruction Caused by Iatrogenic Factors

**DOI:** 10.1155/2018/7174608

**Published:** 2018-01-30

**Authors:** Gregor-Georg Zafiropoulos, Andreas Parashis, Taha Abdullah, Evangelos Sotiropoulos, Gordon John

**Affiliations:** ^1^College of Dental Medicine, University of Sharjah, Sharjah, UAE; ^2^College of Dentistry, Ohio State University, Columbus, OH, USA; ^3^College of Dental Medicine, Mohammed Bin Rashid University of Medicine and Health Sciences, Dubai, UAE; ^4^School of Dentistry, University of Duesseldorf, Duesseldorf, Germany

## Abstract

Dental implant success requires placement after periodontal therapy, with adequate bone volume, plaque control, primary stability, control of risk factors, and use of well-designed prostheses. This report describes the surgical and prosthetic management of a patient with severe iatrogenic periodontal/periimplant bone destruction. *Methods.* A 55-year-old female smoker with fixed partial dentures (FPDs) supported on teeth and implants presented with oral pain, swelling, bleeding, and a 10-year history of multiple implant placements and implants/prosthesis failures/replacements. Radiographs showed severe bone loss, subgingival caries, and periapical lesions. All implants and teeth were removed except implants #4 and #10 which served to retain an interim maxillary restoration. Bone defects were covered with nonresorbable dPTFE membranes. In the mandible, three new implants were placed and loaded immediately with a bar-retained temporary denture. *Results.* Seven months postoperatively, the bone defects were regenerated, and three additional mandibular implants were placed. All mandibular implants were splinted and loaded with a removable overdenture. *Conclusions.* In this case, periimplant infection and tissue destruction resulted from the lack of periodontal treatment/maintenance and failure to use evidence-based surgical and loading protocols. Combination therapy resolved the disease and the patient's severe discomfort while providing immediate function and an aesthetic solution.

## 1. Background

Nowadays, implant-supported restorations are generally accepted as a state-of-the-art treatment option. Many advances in materials and techniques, in surgical and loading protocols, in restorative design as well as a better understanding of the biological/mechanical concepts of osseointegration and of the importance of infection resolution before placement and maintenance, made implants more acceptable by the dental community. Furthermore, appropriate implant treatments are becoming increasingly important also for the general dentists as the number of implants placed per year continues to increase. Gaviria et al. [1] analyzing data of the American Association of Oral and Maxillofacial Surgeons reported that approximately 100,000 to 300,000 dental implants are being placed every year. Also in Germany, the published data showed 200,000 placed implants in the year 2000, and according to statements of scientific societies, the recent number of placed implants is 1.2 million [2].

Periimplantitis, one of the main factors of implant failure, is an inflammatory condition involving the soft and hard tissue surrounding the implant. The 6th European Workshop on Periodontology considered bacterial plaque as the main etiological factor for periimplant tissue damage and also included poor oral hygiene and history of periodontitis as risk indicators [3]. Despite technological, surgical, and material advancements that contribute to enhanced implant survival and/or success, placing dental implants still requires thorough education, training, and continuous professional development in order to acquire the knowledge of which materials, which surgical techniques, which type of loading, and which type of restorations are indicated in every clinical scenario. In other words, implants should be placed by well-trained, qualified clinicians [4].

This report describes the surgical and prosthetic management of a patient with severe iatrogenic periodontal and periimplant bone destruction.

## 2. Case Presentation

A 55-year-old female, smoker (4–6 cigarettes/day), in good general health presented in our clinic in May 2015 with the chief complaint of strong and acute pain in both arches as well as generalized spontaneous bleeding and suppuration (see Case Management). The patient did not consent to intraoral photography at the initial visit. She reported that the same dentist had performed all prior treatments.

### 2.1. Treatment History

**Figure 1:**
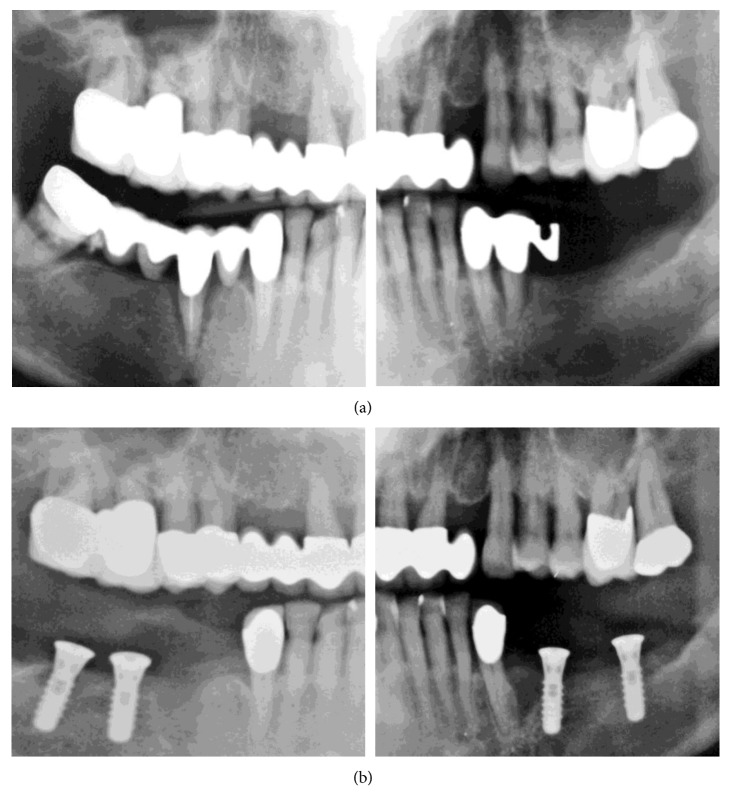
OPGs (original OPG of previous treatment modified for presentation reasons). (a) Before the initiation of previous treatment (January 2004). (b) After mandibular tooth extraction and implant placement (April 2004).

**Figure 2:**
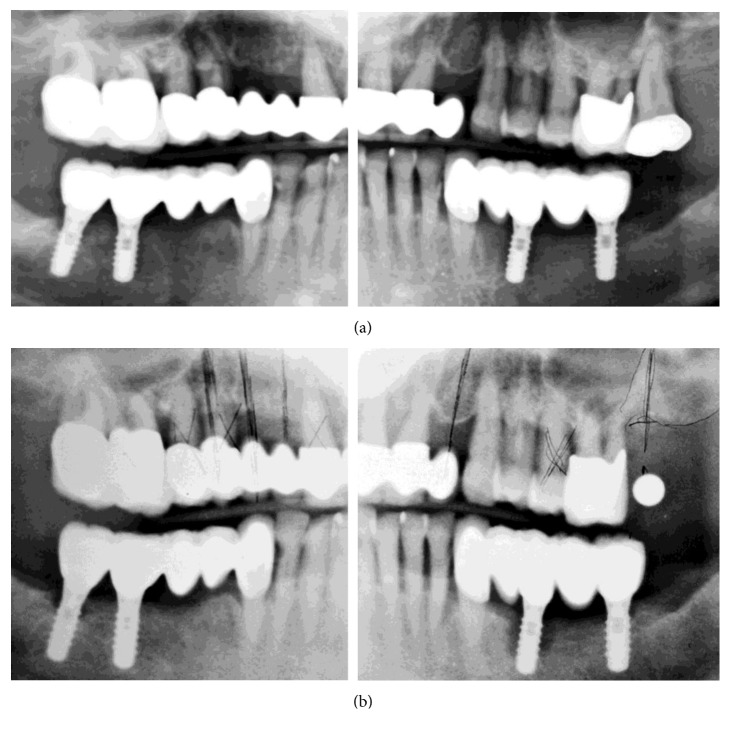
OPGs (original OPG of previous treatment modified for presentation reasons). (a) After mandibular implant loading (July 2004). (b) After extraction of tooth #15 (January 2006).

In January 2004, generalized severe periodontal disease with deep pockets and severe mobility was diagnosed (Figure 1(a)). The patient was not informed about the presence of or need to treat severe periodontitis. In April 2004, teeth #21, #29, and #32 were extracted, and implants were placed in positions #18, #20, #30, and #31/32 (Figure 1(b)). The bone defect at position #21 was not augmented, and no periodontal treatment was performed. In July 2004, the implants were loaded with fixed partial dentures (FPDs) connecting to teeth #22 and #27 (Figure 2(a)). The bone defect at position #21, periimplant bone loss at position #20, and progressing periodontal disease were not treated.

**Figure 3:**
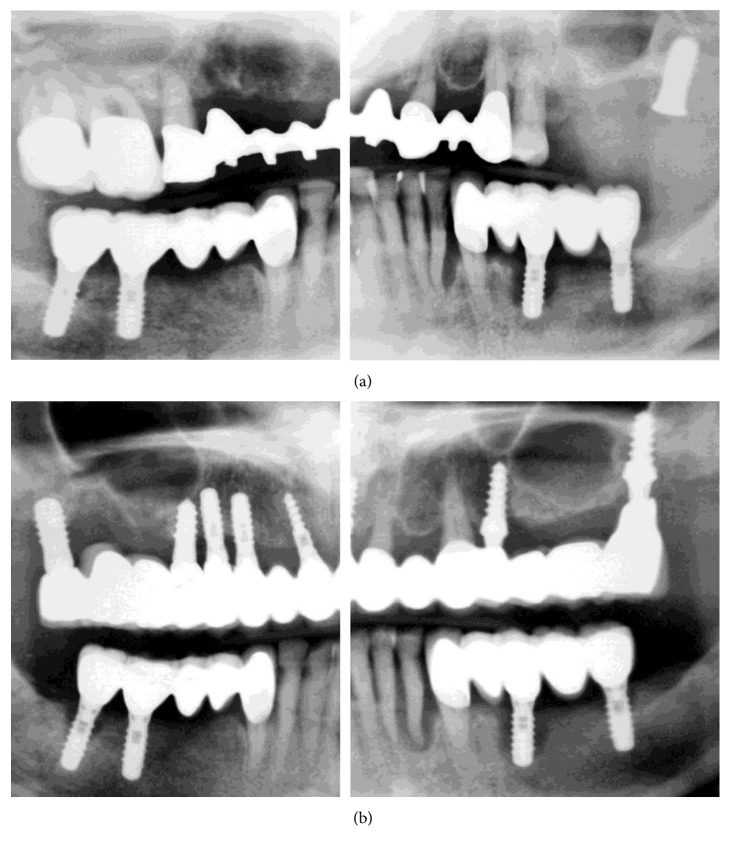
OPGs (original OPG of previous treatment modified for presentation reasons). (a) After extraction of tooth #14 and implant placement in position #15/16 (November 2006). (b) After maxillary restoration (November 2007).

In January 2006, partial healing of extraction socket #21, a bone defect with periapical involvement (#23), and two periimplant defects (#20 and #31; >50% and <50% implant length, resp.) were diagnosed (Figure 2(b)). Tooth #15 was extracted, an implant plan was made (as shown in the orthopantomograph (OPG)), and no further periodontal/periimplant treatment was performed. Between the end of January and October 2006, teeth #5–8, #10, #12, and #15 were extracted; a composite veneered FPD was inserted with teeth #4, #9, and #11 as abutments; and an implant in position #15/16 was placed, but appeared to have only 50% bone contact (Figure 3(a)). No further periodontal/periimplant treatment was performed.

The patient reported visiting the dental office often due to pain, resulting in the fitting of a new maxillary restoration with immediate implant placement and loading in November 2006. The mandibular periimplant defects showed further progression (Figure 3(b)). A new implant in position #15 was placed (compare with implant geometry on Figure 3(a)), tooth #12 was replaced with an implant, and additional implants were placed in positions #1, #4–6, and #8. The new implants had insufficient bone contact; the implant in position #1 had only apical contact with bone. In the subsequent 2 years, the patient complained often about pain and visited the dental office regularly. However, other than superficial cleaning, no periodontal/periimplant treatment was performed.

**Figure 4:**
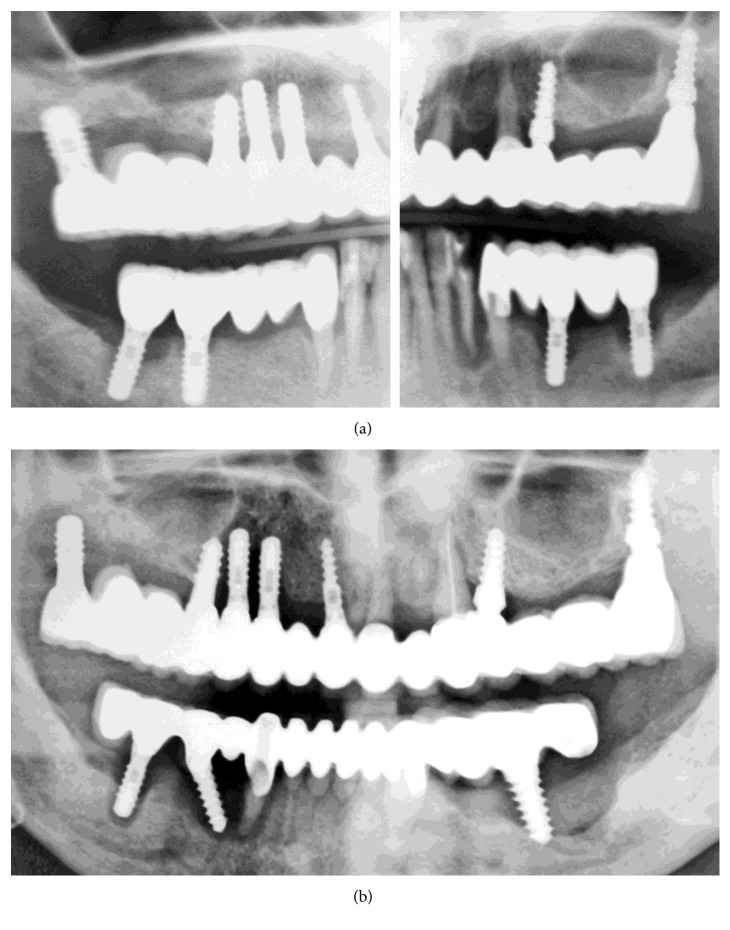
OPGs. (a) Further progression of bone loss on November 2009 (not modified radiograph taken during current treatment). (b) At initial examination in June 2015 (not modified radiograph taken during current treatment).

An OPG taken in November 2009 demonstrated further progression of bone loss (Figure 4(a)). The patient reported that the dentist in 2010 removed the mandibular FPDs, implants, and the majority of teeth and inserted another fixed restoration with immediate placement and loading, connecting the three implants with teeth #22 and #27. No OPG showing this treatment or follow-up were available. The patient visited the dental office regularly for cleaning and complained of new pain. In 2015, she was referred for periodontal consultation. Comparison of Figures 4(a) and 4(b) shows that the mandibular implants were explanted, and three new implants were placed and loaded.

### 2.2. Case Management

Comprehensive dental and periodontal examinations were performed, and an OPG was made (Figure 4(b)). All maxillary and mandibular implants and teeth showed radiographic severe bone loss, and teeth #9, #11, and #27 additionally showed subgingival caries and periapical lesions. Periimplant pockets were 6–10 mm deep with spontaneous bleeding, soft-tissue swelling, and pain on palpation.

After receiving oral and written descriptions of the proposed treatment, including surgical procedures, the patient provided written informed consent. To address the acute condition, mandibular periimplant abscesses were drained through the pockets, and clindamycin (800 mg/day) was prescribed, due to the patient's reported allergy to penicillin. The patient's file and radiographs were retrieved from her former dentist.

**Figure 5:**
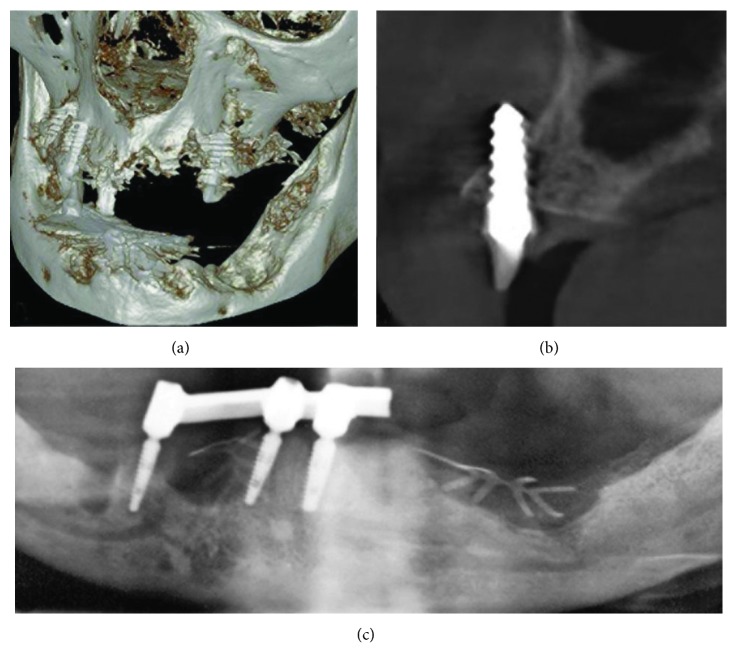
(a) Volumetric 3D representation of hard tissue and maxillary implants, taken during mandibular surgery, demonstrating large bone defects and loss of buccal bone plate in the maxilla. (b) Axial CBCT section of the maxilla showing misplaced implant #4. (c) OPG section showing bar retained on the remaining three mandibular implants (not modified radiograph taken during current treatment).

**Figure 6:**
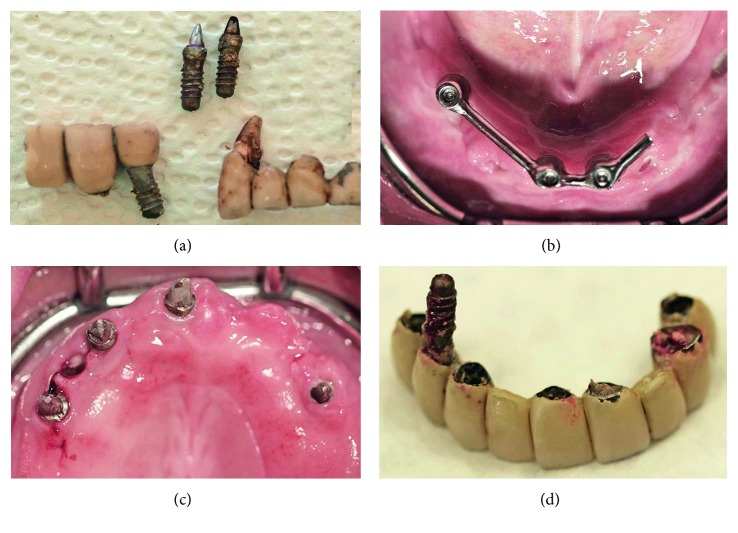
(a) Explanted mandibular implants and tooth #27. (b) Clinical view of the mandible 4 weeks postoperatively, before membrane removal. (c) The maxilla after FPD removal. (d) Explanted maxillary implant during FPD removal.

All mandibular and maxillary implants and teeth were removed, except implants #4 and #10 which served to temporarily retain an interim maxillary restoration. During surgery and after removal of the mandibular teeth and implants and cleaning of the bone defects, a cone beam computed tomograph (CBCT) was made (Figures 5(a) and 5(b)). The extraction sockets and periimplant bone defects were cleaned, and gentamicin-loaded collagen fleeces (Jason; Botiss Biomaterials, Zossen, Germany) were placed in the defects [5]. Subsequently, the defects were covered with nonresorbable dense polytetrafluoroethylene membranes (dPTFE; Cytoplast Ti-250; Osteogenics Biomedical, Lubbock, TX, USA) without additional bone grafting, as previously described [6]. Implants (K3Pro rapid; 3.5 mm diameter, 11 mm length: Argon Dental, Bingen/R, Germany) were placed in positions #24, #26, and #30 and loaded the same day with a bar-retained removable temporary denture. The membranes were removed 4 weeks postoperatively (Figures 5(c), 6(a), and b6(b)). The bar was milled of type 3 CrCo alloy (ZENOTEC NP; Wieland, Pforzheim, Germany), a metal base was constructed, and elastic plastic clips (Preci Matrice, CEKA, Waregem, Belgium) were used to retain the base over the bar.

**Figure 7:**
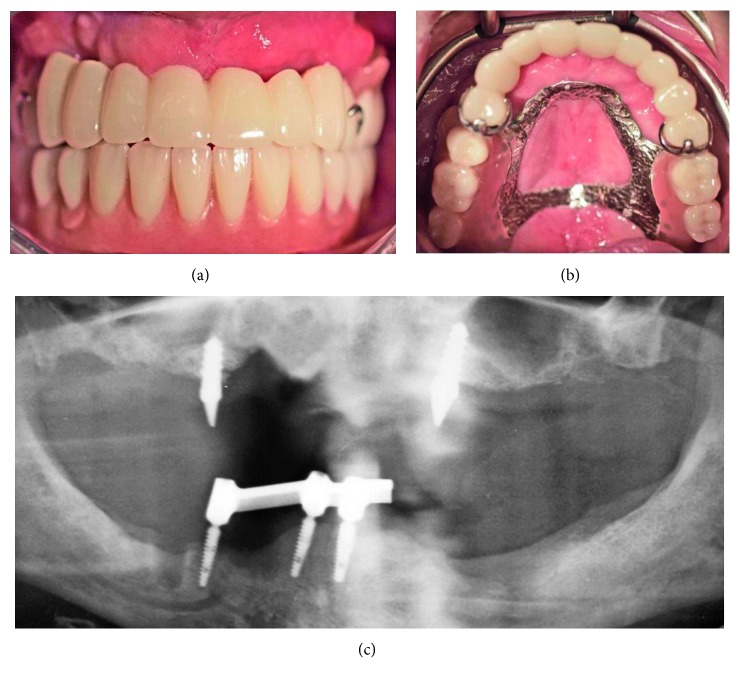
(a, b) Maxillary temporary rehabilitation with FPD retained on implants #4 and #10 and removable denture for the molar areas. (c) OPG 4 weeks after surgery with the mandibular overdenture (not modified radiograph taken during current treatment).

On the same day, all remaining maxillary teeth and implants, except #4 and #10, were extracted, periimplant lesions on #4 and #10 were treated (Figures 6(c) and 6(d)), and the maxilla was temporarily restored with a milled FPD fixed on the implants #4 and 10 using provisional cement (Implant Provisional; Alvelogro Inc., Snoqualmie, WA, USA) and a removable partial denture for the molar areas (Figure 7).

**Figure 8:**
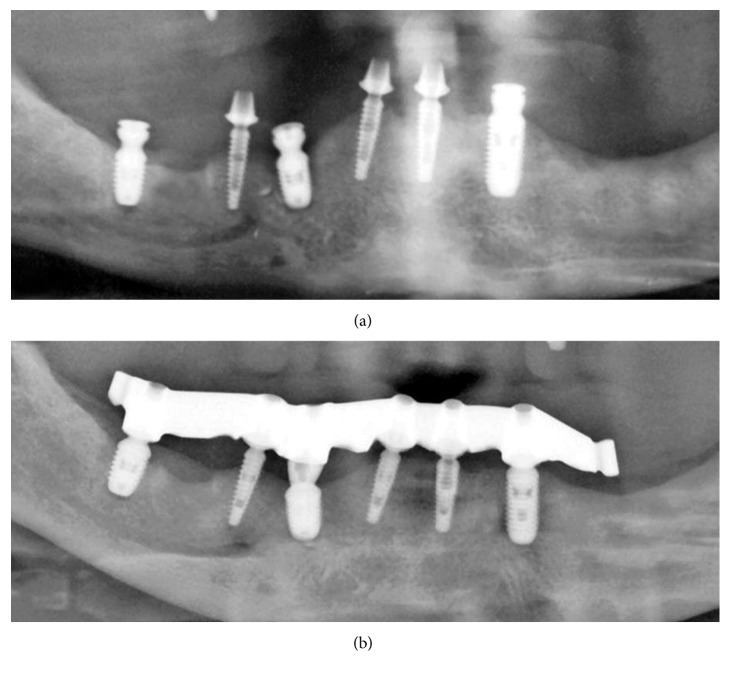
Mandibular OPG sections eight months postoperatively (not modified radiograph taken during current treatment). (a) After placement of three additional implants. (b) After bar mounting.

**Figure 9:**
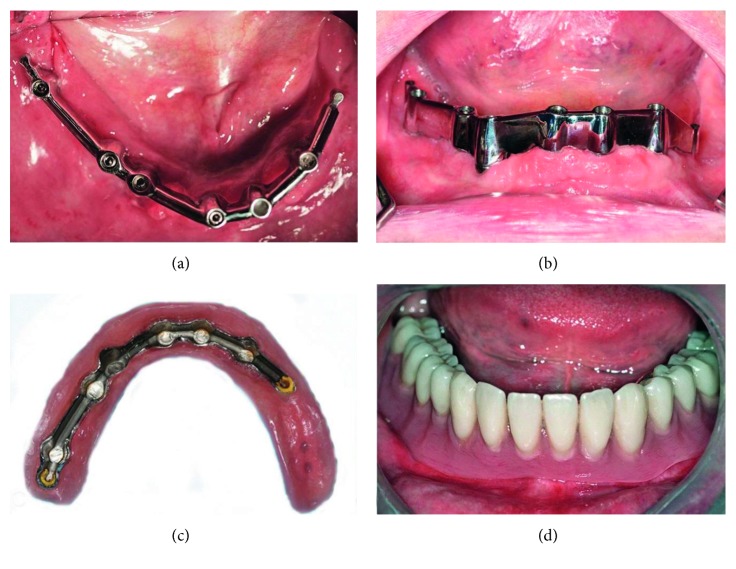
Clinical view of the final mandibular bar restoration after bar mounting. (a) Occlusal view after one week. (b) 30 days after loading. (c) Denture's base. (d) Mandibular denture in situ.

Seven months postoperatively, the bone defects were regenerated, and three additional mandibular implants were placed in positions #22, #28, and #31/32 (K3Pro rapid; 4.5 mm diameter, 9 and 11 mm lengths, Argon Dental) (Figure 8(a)). All six mandibular implants were splinted with a milled bar and loaded as described previously (Figures 8(b) and 9).

## 3. Discussion and Conclusions

In the present case report, the surgical and prosthetic management of a patient with multiple teeth and implants with severe bone loss and a hopeless prognosis due to iatrogenic factors, with extractions, bone regeneration, immediate implant placement, and insertion of prosthesis, is discussed. The patient was treated by the same dentist in the period between January 2004 and April 2015.

Dental implant success and survival requires placement after periodontal therapy, adequate bone volume/quality, nontraumatic surgery, primary stability, control of risk factors, and use of well-designed prostheses. In addition, adequate plaque control and regular maintenance (infection control) and early detection and treatment of periimplant inflammation are also important for long-term success [7–14].

Implants in patients treated for periodontal disease are associated with higher incidence of biologic complications and lower survival rates than those in periodontally healthy patients, and severe forms of periodontal disease are associated with higher rates of implant loss [7]. Several studies and systematic reviews have concluded that, before implant placement, any existing periodontal disease must be treated, periodontally susceptible patients have a higher risk of developing periimplantitis, and in cases with periodontally compromised teeth with probing depths >5 mm, the colonization of implants by periodontal pathogens is possible and could be considered as a risk factor. Furthermore, there is evidence that bone loss in periodontitis patients will progress in the absence of periodontal treatment [7–11].

The importance of an accurate diagnosis and an appropriate treatment plan are essential in management of periodontal disease [7]. Based on the radiographs and the information obtained by the patient's file submitted by the previous dentist, one can conclude that she was suffering from severe chronic periodontal disease which was left untreated. In addition, the progression of periimplant inflammation was ignored and not treated although periimplant bone destruction was visible on the regularly taken radiographs. The patient reported regular oral hygiene appointments in the dental office but only supragingival debridement was performed.

Currently, there is not enough focus on the prevention of periimplant diseases, as compared to periodontal maintenance [7, 13]. It is well known that, in periodontitis susceptible patients treated with dental implants, residual pockets represent a significant risk for the development of periimplantitis and implant loss. Moreover, patients in supportive periodontal treatment developing reinfections are at greater risk for periimplantitis and implant loss than periodontally stable patients [14].

**Figure 10:**
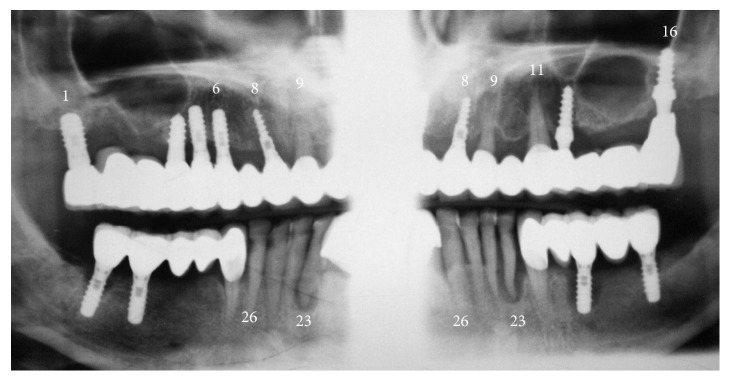
Original not modified OPG of previous treatment with significant distortion. Please compare with Figure 3(b). Double representation of teeth and implants is indicated.

An additional finding, after examining the patient's file, was the absence of accurate radiographs of diagnostic quality or the use of surgical guidance for implant placement. The used OPGs were of extremely poor quality, with a double representation of teeth and implants and significant distortion (Figures 1–3, 4(a), and 10). Thus, they had to be processed with a raster graphics editor (Photoshop Elements 15, Adobe Systems, Munich, Germany) for presentation reasons (Figures 1–3 and 4(a)). An accurate diagnosis was not possible on these OPGs, and they should not have been used for surgical planning. Although the use of two- or three-dimensional radiography in all or selected implant cases [15] and the routine use of different types of surgical guides or navigated implantology [16] is still a debate, the use of minimal appropriate diagnostic tools and procedures as well as medical and dental standards is mandatory for a successful result after implant placement.

Another treatment modality, which was repeatedly applied in the presented case, was immediate implant placement and loading in infected and compromized periodontal tissues as well as the connection of teeth and implants. Furthermore, the restorations did not fit on the abutments (Figure 4(b)). These could be additional factors for teeth and implants loss. In the present case, an immediate implant placement and eventually loading could be possible, only by following established rules and clinical protocols as well as guidelines from the scientific literature. However, the lack of knowledge has led to a disaster [4, 17, 18].

Combination therapy resolved the disease and the patient's severe discomfort while providing immediate function and an aesthetic solution. Patient's rehabilitation was achieved by elimination of the infection, bone regeneration, and implant placement. In the mandible, three implants were placed during the first surgery, splinted and loaded with an overdenture, restoring function, and aesthetics. In addition, the bar-retained mandibular overdenture protected the augmented areas from pressure during the healing period. In the maxilla, implants were removed, periimplant lesions in the remaining two implants were treated, and an aesthetic and functionally acceptable long-term provisional restoration was fabricated.

The long-term periodontal and periimplant infection and tissue destruction presented in this case resulted from lack of periodontal and periimplant treatment as well as maintenance and failure to use evidence-based diagnostic, surgical, and restorative procedures. Combination therapy resolved the disease and the patient's severe discomfort while providing immediate function and an aesthetic solution.
